# Building Capacity in Crisis: Evaluating a Health Assistant Training Program for Young Rohingya Refugee Women

**DOI:** 10.3390/ijerph23010127

**Published:** 2026-01-20

**Authors:** Nada Alnaji, Bree Akesson, Ashley Stewart-Tufescu, Md Golam Hafiz, Shahidul Hoque, Farhana Ul Hoque, Rayyan A. Alyahya, Carine Naim, Sulafa Zainalabden Alrkabi, Wael ElRayes, Iftikher Mahmood

**Affiliations:** 1 Hope Foundation for Women & Children of Bangladesh, Ramu 4700, Bangladesh; alnaji.nada@gmail.com (N.A.); mghafiz.bd@gmail.com (M.G.H.); shahidcox@gmail.com (S.H.); farhana.hoque17@gmail.com (F.U.H.); iumahmood@gmail.com (I.M.); 2 Faculty of Social Work, Wilfrid Laurier University, Waterloo, ON N2L 3C5, Canada; 3 Faculty of Social Work, University of Manitoba, Winnipeg, MB R3T 2N2, Canada; ashley.stewart-tufescu@umanitoba.ca; 4 Saudi Center for Organ Transplantation, Riyadh 12823, Saudi Arabia; rayyan.alyahya@gmail.com; 5 Independent Researcher, Beirut, Lebanon; carinenaim@gmail.com; 6 Faculty of Health and Medicine, Lancaster University, Lancaster LA1 4YW, UK; s.alrkabi@lancaster.ac.uk; 7 Department of Health Services Research and Administration, College of Public Health, University of Nebraska Medical Center, Omaha, NE 68198, USA; wael.elrayes@unmc.edu

**Keywords:** refugee health, capacity building, Rohingya crisis, economic empowerment, program evaluation, child and maternal health care

## Abstract

Background: The Rohingya refugee crisis is one of the largest humanitarian emergencies of the 21st century, with nearly one million Rohingya residing in overcrowded camps in southern Bangladesh. Women and children face the greatest vulnerabilities, including inadequate access to education and healthcare, which exacerbates their risks and limits opportunities for personal and community development. While international organizations continue to provide aid, resources remain insufficient, particularly in maternal and child healthcare, highlighting the urgent need for sustainable interventions. Objectives: The Hope Foundation for Women and Children in Bangladesh launched a pilot project for the Health Assistant Training (HAT) program to address critical gaps in healthcare and education for the Rohingya community. This nine-month training program equips young Rohingya women with essential knowledge and skills to support maternal health services in both clinical and community settings. Design: We conducted a qualitative evaluation of the HAT Program to explore its acceptance and anticipated benefits for both participants and the community. Methods: The research team used semi-structured interviews, focus groups, and field observations to explore the HAT Program’s impact on young Rohingya women and their community. They analyzed data through thematic analysis, developing a coding framework and identifying key themes to uncover patterns and insights. Results: The results were categorized into four themes: (1) community acceptance of the HAT Program, (2) the HAT Program’s impact on the health assistant trainees, (3) the impact of the HAT Program on the community, and (4) the potential ways to expand the HAT Program. Conclusions: This research underscores the program’s impact on improving healthcare access, enhancing women’s empowerment, and promoting community resilience. By situating this initiative within the broader context of refugee health, education, and capacity-building, this research highlights the HAT program’s potential as a replicable model in Bangladesh and in other humanitarian settings.

## 1. Introduction

The Rohingya, a predominantly Muslim ethnic group from Myanmar’s Rakhine State, have faced decades of persecution, including denial of citizenship under Myanmar’s 1982 Citizenship Law and systematic violence that culminated in what the United Nations has described as “a textbook example of ethnic cleansing” in 2017 [1]. Since 2017, over one million Rohingya refugees have fled to neighboring Bangladesh, where they reside in the world’s largest refugee settlement [2].

Women and children comprise the majority of displaced Rohingya, many of whom bear the dual burden of trauma and vulnerability in precarious camp conditions, which include overcrowding, poor sanitation, and limited infrastructure [3,4]. Rohingya women and children face heightened risks due to limited access to health services in the camps. In particular, maternal health remains a critical concern, with remarkably high maternal mortality rates (about 400 deaths per 100,000 live births) that are more than double Bangladesh’s national average and among the highest globally [3]. Agarwal et al. [3] attribute this high maternal mortality rate to “the three delays”, which include delays in care-seeking, reaching facilities, and receiving appropriate care. Gendered cultural norms, including *purdah* (a system of sex segregation prevalent in some Muslim cultures), restricted mobility, limited decision-making power, and reliance on home births further shape the population’s high maternal mortality rate [3,5]. These risks are further compounded by the high prevalence of child marriage in the camps, where early marriage is often perceived as a protective strategy amid insecurity, yet is associated with early and closely spaced pregnancies, limited reproductive autonomy, and low contraceptive use among Rohingya adolescent girls [6].

Recent evidence from the Rohingya camps highlights the central role of maternal psychosocial wellbeing in shaping caregiver capacity, care-seeking behaviors, and child health outcomes. For example, a randomized cluster trial of Baby Friendly Spaces in the camps demonstrated that integrating psychosocial support into routine health and nutrition services can significantly improve maternal distress, functioning, and wellbeing [7]. This emphasis on integrated care is particularly salient in the Rohingya context, where distress is shaped by chronic stressors and often expressed through somatic and functional complaints, positioning frontline health workers as critical entry points for psychosocial support [8]. Complementary evidence from midwife-led birthing centers in the camps further underscores the importance of respectful, female-provider-led care embedded within coordinated systems of service delivery, where trust, communication, and supervision play critical roles in women’s engagement with maternal health services [9]. The *Joint Response Plan for Rohingya Humanitarian Crisis in Bangladesh* [10] similarly emphasizes community health education and behavior change communication through Community Health Workers, including expanding livelihood opportunities in roles such as health promotion to strengthen self-reliance and resilience within the camps. Yet despite these efforts to improve healthcare infrastructure, the demand far exceeds available resources, resulting in persistent gaps in access and continuity of care.

The challenges for Rohingya women and children extend beyond healthcare access. For years, government policies have severely restricted formal education for Rohingya children, including a ban on the use of the Bangladesh national curriculum in the camps, only allowing informal education through NGOs [11,12,13,14]. While recent efforts from international actors such as UNICEF, Save the Children, and partners have led to a 7% increase (from 17% to 24%) in Rohingya girls attending secondary education classes in the 2023–2024 school year [15,16], a significant number remain without access to educational programs, reflecting cultural, logistical, and systemic barriers that restrict young Rohingya women from accessing learning and vocational opportunities [17]. While initiatives such as the Myanmar Curriculum Pilot (MCP) introduced in 2020 aimed to formalize education for refugee children, significant challenges remain, including limited resources and deeply entrenched cultural norms that deprioritize education for girls [18]. These barriers not only limit personal growth but also hinder community development, as women play a critical role in shaping health and wellbeing within their families.

Despite international humanitarian efforts, conditions in the camps remain dire, with limited access to healthcare, education, and livelihoods, while the prospects for safe repatriation to Myanmar remain bleak due to ongoing persecution and lack of citizenship rights [1,14,19,20]. Recent cuts in humanitarian aid, driven largely by drastic changes in U.S foreign-assistance policy, have further exacerbated these conditions, leaving these refugees increasingly vulnerable and deepening the crisis [21]. The Rohingya refugee crisis, therefore, represents one of the largest humanitarian emergencies of the 21st century, underscoring the urgent need for global solidarity and sustainable solutions to address the plight of the Rohingya.

## 2. The Health Assistant Training (HAT) Program

Against this backdrop, the Hope Foundation for Women and Children in Bangladesh (henceforth referred to as Hope Foundation) launched the Health Assistant Training (HAT) Program, as a pilot initiative aimed at addressing critical gaps in healthcare and education in the camps. The nine-month HAT Program was launched in 2023 to equip young Rohingya women with essential knowledge and skills to support maternal health services in both clinical (e.g., field hospital) and community settings in the camps. HAT trainees were recruited through a purposive, community-based process led by the Hope Foundation in close coordination with camp leadership. Community leaders from blocks surrounding the Hope Field Hospital were informed about the program objectives and invited to nominate young women. Eligibility criteria were intentionally flexible to reflect the educational realities of the camps and included basic literacy (ability to read and write), interest in health-related work, and family consent to participate. Following nomination, candidates were invited to an orientation session at the hospital where the program structure, time commitment, and expectations were explained. Final selection was based on availability, willingness to complete the full training period, and perceived ability to engage meaningfully with staff and classmates. This recruitment approach prioritized community acceptance and feasibility rather than representativeness, consistent with the pilot and exploratory nature of the program. As part of their enrollment in the HAT Program, trainees received standard program supports, including daily meals during training days, a small stipend, uniforms, and learning materials.

The program followed Competency-Based Learning with periodic assessments. Training was divided into three segments. The first three months covered foundational subjects such as basic communicative English, community and primary health, introduction to basic medical equipment, and fundamentals of maternal and child healthcare and family planning. The second segment focused on advanced concepts in maternal and child healthcare, medical ethics and health promotion, basic infection prevention and control, and the roles and responsibilities of a healthcare assistant. The final segment was a three-month internship providing hands-on experience under supervision.

While the HAT Program has the potential to improve maternal health services and empower women, there has yet to be an evaluation of the program among the trainees within their communities. Therefore, this evaluation aimed to understand the acceptance and anticipated benefits of the HAT Program for both trainees and the broader community. Through an analysis of qualitative data from trainees, community leaders, and other stakeholders, this manuscript underscores the program’s impact on improving healthcare access, enhancing women’s empowerment, and promoting community resilience. By situating this initiative within the broader context of refugee health and education, this work highlights the HAT program’s potential as a promising model in Bangladesh and in other humanitarian contexts.

From a theoretical and methodological perspective, this study is informed by evidence-based and evidence-informed program evaluation frameworks that emphasize capacity building within marginalized and crisis-affected communities. Research across humanitarian and refugee settings consistently demonstrates that training community members as frontline health workers is a critical strategy for addressing service gaps, strengthening trust, and overcoming linguistic and cultural barriers to care [22,23,24]. Capacity-building approaches, specifically those embedded within community health worker and workforce-development models, prioritize the diffusion of essential knowledge and skills through locally trusted actors, thereby enhancing both program acceptability and sustainability [25,26]. For example, evidence from integrated maternal-child interventions in the Rohingya camps suggests that capacity building is most effective when accompanied by structured supervision, provider support, and attention to implementation fidelity, particularly in low-intensity, community-based programs [7]. Within refugee contexts such as Bangladesh, these approaches are especially salient given restrictions on formal employment, shortages of skilled personnel, and mistrust of external service providers.

Although the HAT Program was not formally structured as a training-of-trainers initiative, it draws on similar principles by equipping young Rohingya women with transferable health knowledge and skills that can be shared within households and the broader community. In humanitarian settings where formal professional pathways are restricted, such capacity-building models are often a pragmatic strategy for extending reach and impact [27,28]. Anchoring this evaluation within a capacity-building and empowerment-oriented framework therefore provides a lens for examining not only individual skill acquisition, but also anticipated community-level effects, including improved access to care, enhanced health literacy, and strengthened social cohesion.

## 3. Methods

This evaluation utilized a qualitative approach to evaluate the impact of the HAT Program on participants and their surrounding community. The primary objectives were to explore the cultural nuances embedded in the program, assess the empowerment of young Rohingya women via the HAT trainees’ experiences, and examine the broader societal implications of the initiative. Consistent with empowerment-oriented and capacity-building program evaluation approaches, this qualitative evaluation focused on perceived acceptability, anticipated benefits, and pathways for community-level knowledge diffusion. As such, this study constitutes a formative and exploratory program evaluation, rather than an outcome or effectiveness evaluation.

### 3.1. Evaluation Team

Two evaluation team members—one male (the fifth author) and one female (the sixth author)—were responsible for recruitment and data collection. Both team members were from Bangladesh, with the male team member originating from the local Cox’s Bazar region, where the local dialect is Chittagonian. While Chittagonian is linguistically distinct from Rohingya, it is closely related and widely used as a *lingua franca* in the refugee camps. Both interviewers routinely worked in Rohingya-speaking settings and were familiar with Rohingya-specific vocabulary, expressions, and cultural norms, which facilitated effective communication and rapport-building during data collection.

Both team members had graduate degrees and over five years of experience in public health research in humanitarian settings. At the time of the evaluation, they were employed as public health researchers at Hope Foundation. Both completed the *Canadian Tri-Council Policy Statement (TCPS-2): Ethical Conduct for Research Involving Humans* certification, as well as a 23 h online training in qualitative research methods. In addition, the first author conducted three specialized trainings with the evaluation team focused on qualitative interviewing, ethical engagement in refugee contexts, and reflexive monitoring and evaluation practices.

### 3.2. Participants

The study included three participant groups: HAT trainees, community leaders (known as *majis*), and representatives from non-governmental organizations (NGOs) working in the camps (see Table 1). All HAT trainees were female Rohingya aged 14–19 years, reflecting the program’s focus on training young women to support maternal and child health services. This age range also reflects the broader social context of the camps, where adolescent girls face heightened risks of early marriage and early pregnancy, shaping both their health trajectories and opportunities for education and skill development [6]. Community leaders interviewed were *majis*, or male community representatives appointed to act as intermediaries between refugee populations and camp authorities and humanitarian actors [29]. In the Rohingya camps, *majis* play a central role in information dissemination, coordination of assistance, and liaison with NGOs, making them key stakeholders for understanding community perceptions of health and education initiatives. NGO participants were professionals working in health, education, or humanitarian coordination roles relevant to the implementation and sustainability of the HAT Program.

### 3.3. Research Ethics

The evaluation team consulted with the University of Nebraska Medical Center’s (UNMC) Institutional Review Board (IRB), which determined that this project constituted program evaluation and monitoring activities and therefore did not meet the definition of human subjects’ research under 45 CFR 46.102. Nonetheless, the evaluation was conducted in accordance with rigorous ethical procedures consistent with international standards for ethical practice in humanitarian settings. For Health Assistant Training (HAT) Program trainees, parental consent was obtained at the time of program enrollment, covering both participation in the training program and any related program monitoring and evaluation activities. All participants completed a culturally appropriate verbal informed consent process prior to participation in any evaluation activities. Participants were informed of the purpose of the evaluation, the procedures involved, the voluntary nature of participation, and their right to decline or withdraw without any impact on services, training, or support received through the Hope Foundation. Verbal consent was documented only after participants’ understanding was confirmed, and additional consent was obtained for audio-recording interviews and focus group discussions. No incentives were provided for participation in the evaluation. No identifiable personal data were collected, no images of participants are included in the manuscript, and all findings are reported in a manner that protects participants’ privacy, confidentiality, and dignity, ensuring that individuals cannot be identified directly or indirectly.

### 3.4. Methods of Data Collection

To gather comprehensive insights, the two evaluation team members conducted semi-structured interviews, focus group discussions, and observational field notes. These approaches helped create a detailed understanding of the HAT Program’s outcomes and potential for refinement and scalability. The evaluation team facilitated two focus group discussions with nine of the health assistant trainees (ages 14–19) who were currently in the program to delve deeper into their aspirations, expectations, and experiences. All HAT trainees were invited to participate in the evaluation, and all agreed to participate. The evaluation team also conducted eight 30–40 min semi-structured interviews with *majis* from neighboring blocks around the Hope Foundation Field Hospital to explore their perceptions of the HAT Program’s role in empowering young women and its broader societal impacts. All the *majis* invited agreed to participate in the evaluation. Semi-structured interviews with HAT trainees and *majis* took place in private rooms at the Hope Field Hospital. Non-participants were not allowed in the room during focus group discussions with trainees or semi-structured interviews with *majis*. Semi-structured interviews and focus group discussions were conducted in the language preferred by participants (Rohingya, Chittagonian, Bangla, or English). Additionally, the evaluation team interviewed representatives from the Hope Foundation and other NGOs working with Rohingya refugees. These interviews provided valuable insights into the program’s feasibility and sustainability, as well as potential employment opportunities for HAT graduates.

Our interview guides were not formally validated because they were designed to be flexible, adaptive tools rather than rigid, standardized instruments. The evaluation team refined the semi-structured interview guide iteratively based on initial interviews with participants, emerging themes, and participant responses, making formal validation impractical. Additionally, our evaluation prioritized depth, nuance, and open-ended inquiry, meaning that the effectiveness of our interview guide was assessed through expert review and ongoing refinement.

For semi-structured interviews with NGO stakeholders/representatives conducted in English, the evaluation team member took meticulous notes. For semi-structured interviews and focus group discussions conducted in Rohingya, sessions were recorded, transcribed, and translated into English by the evaluation team. This approach ensured the integrity and authenticity of the data while making it accessible to English-speaking team members. Written transcripts were not returned to participants due to low literacy levels.

The first author also took observational field notes throughout the evaluation, which were also included in the analysis. These notes captured contextual observations related to the training environment, participant interactions, and program implementation.

### 3.5. Data Analysis

For sessions conducted in Rohingya or Chittagonian, audio recordings were first transcribed in the original language used during data collection. These transcripts were then translated into English by the same bilingual team member who conducted the interviews, allowing for contextual interpretation rather than literal word-for-word translation.

To address potential challenges related to linguistic nuance and meaning, translations were reviewed collaboratively within the evaluation team to clarify interpretations, resolve ambiguities, and ensure that key concepts, expressions, and participant perspectives were accurately represented. English-language transcripts were then reviewed through repeated readings by the broader team.

Data were analyzed thematically to identify patterns and shared meanings across participant groups. The two team members who conducted the interviews (the fifth and sixth authors) completed the initial coding, after which three additional team members who were not involved in data collection independently read, re-read, and recoded the data.

The first author developed the final coding framework by integrating pre-established codes from the interview guide with emergent codes identified during analysis. Related codes were grouped into themes and subthemes aligned with the study objectives. Representative quotations were selected to illustrate findings and ensure participants’ voices remained central.

Observational field notes were not analyzed as a separate dataset and therefore do not appear as standalone findings. Instead, they were used to support data familiarization, inform interpretation, and aid triangulation of themes emerging from semi-structured interviews and focus group discussions. In cases where observational insights informed interpretation, they were integrated implicitly into the narrative rather than presented as independent evidence.

Data triangulation across health assistant trainees, community leaders, NGO representatives, and observational field notes enhanced analytical rigor and strengthened the credibility of findings, thereby enabling an assessment of both individual-level and community-level capacity-building outcomes.

## 4. Results

The results were categorized into four themes: (1) community acceptance of the HAT Program, (2) the HAT Program’s impact on the health assistants, (3) the impact of the HAT Program on the community, and (4) the potential ways to expand the HAT Program.

### 4.1. Theme 1: Community Acceptance of the HAT Program

This theme explores the acceptance of the HAT Program by the community, with a focus on two subthemes: (a) cultural views on women’s participation in vocational training, and (b) the potential impact on gender dynamics.

*(a)*  *Cultural views on women’s participation in vocational training*

The health assistants described the decision-making process for joining the HAT Program, emphasizing the role of family discussions in shaping their decisions. They mentioned that their participation was openly discussed with family members, including their father and mothers, who were supportive and actively encouraged them to join the HAT Program. For example, one trainee explained, “I discussed the issue with my family, they agreed to participate in this program” (Participant 6). Similarly, another trainee said, “All my family members agreed to let me take part in this program” (Participant 5).

In addition to family support, community leaders expressed their support for the young women participating in the HAT Program. However, they emphasized the importance of the young women adhering to community norms and Islamic traditions while undergoing training outside their homes. For example, one community leader explained that men and women “…should train separately as our society does not accept joint training” (Leader 1). This was confirmed during discussions with the evaluation team, who work closely with the Rohingya community; they emphasized the importance of maintaining the modesty and safety of the health assistants as a critical factor in ensuring the program is accepted by the community and aligned with cultural expectations. They highlighted measures such as wearing modest uniforms, ensuring the trainees return home before sunset, and providing transportation for those from distant camps as essential for sustaining the program and maintaining community trust.

*(b)*  *Potential impact on gender dynamics*

Data from the HAT Program trainees revealed optimism that their participation could positively influence gender dynamics by promoting women’s empowerment, challenging conservative norms, and fostering gradual societal change. The program equips young Rohingya women with skills that can lead to meaningful employment, which HAT Program trainees believed could elevate their status in this traditional, male-dominated hierarchical society where a female (i.e., a daughter) is often perceived as a financial burden on a family. One health assistant expressed optimism, stating, “If job opportunities are created for females, it will improve women’s status in the society” (Participant 1). This perspective reflects a shared sentiment among the trainees that participation in the HAT Program could shift societal perceptions about women and their roles, with one participant in the focus group discussion adding, “It will affect people’s perception about girls and women” (Participants).

Community leaders, often gatekeepers and enforcers of cultural norms and expectations, also acknowledged the HAT Program’s potential to create positive shifts in gender dynamics in the camps. While the Rohingya community is deeply conservative, leaders noted the influence of new opportunities on traditional practices. As one leader observed, “The Rohingya community is more conservative than Bangladeshi. But such a program could change the dynamics between men and women in the Rohingya community gradually” (Leader 5). Another leader highlighted the subtle yet transformative changes taking place within the community, saying:

Our Rohingya people were very conservative. We are refugees in this place. Girls and boys were engaged in various activities in this place. Thus [the HAT Program] is changing the mentality of isolating women in the home. (Leader 1)

These insights emphasize that the HAT Program is not just about training women for health-related roles but also about fostering a gradual shift in gender norms and expectations.

The potential integration of women into the workforce through the HAT Program marks a significant step toward challenging the social isolation and restrictions often imposed on women in conservative communities such as the Rohingya. By empowering women to contribute economically and socially, the program can dismantle traditional barriers and create pathways for broader societal acceptance of gender equality. Evidence from similar empowerment initiatives in other refugee and conservative settings indicates that when women gain access to education and employment, it often leads to reduced gender inequities, increased community-wide acceptance of women in leadership roles, and improved family dynamics [30,31].

### 4.2. Theme 2: Impact of the Program on the Health Assistants

Theme 2 explores the multifaceted effects of the HAT Program on the health assistants themselves, focusing on three key areas: (a) education and skills development, (b) empowerment and social status, and (c) contributions to income and livelihoods.

*(a)*  *Education and skills development*

Participants described how the HAT Program equips them with practical clinical skills to support mothers and children during the prenatal and perinatal period. Trainees described how they gain valuable knowledge about maternal-child health, with one participant noting, “We will be able to understand the importance of antenatal visit every month by pregnant women” (Participant 2). This education enables women to promote preventive care and improve health outcomes for mothers and infants and may improve health outcomes for care recipients in the camps. Additionally, the program provides hands-on training in essential medical procedures. For example, another participant shared, “I will be able to help others, by learning how to give injection and appropriate medication[s]” (Participant 4). These practical skills not only prepare trainees for employment in healthcare roles but may also enable them to address critical health needs within the Rohingya community, fostering a cycle of wellbeing within the community in the camps. In this way, building individual capacity can lead to broader societal benefits and may even improve healthcare access and maternal and child health outcomes in the camps while contributing to the trainees’ economic and social mobility.

*(b)*  *Empowerment and social status*

The data indicate that the HAT Program can empower them by enhancing their knowledge and skills, ultimately improving their social standing within their families and communities. As one trainee expressed, “My position in society will be boosted and my family [will] value me more” (Participant 2-Focus Group 2), highlighting how the program fosters both self-worth and familial recognition. Additionally, a community leader emphasized that, “With the power of knowledge, the girls feel stronger and more important within society” (Leader 5), reinforcing the idea that education equips women with confidence and respect. By providing essential healthcare training, the HAT Program not only improves women’s autonomy and self-actualization but also strengthens their role as valuable contributors to their families and their communities.

*(c)*  *Contributions to income and livelihoods*

Participants noted that the HAT Program offered Rohingya women a critical opportunity to gain certification, enabling them to work and support their families, in a context where such opportunities are extremely limited. As one trainee stated, “We would like to get a certificate after completing this program and after that we will be able to get validation for working here. Also, we can work in Myanmar once we go back there” (Participants-Focus group 1). This certification not only provides a pathway for employment in healthcare roles within the camps, but also holds the potential for future work in Bangladesh or Myanmar, should repatriation become viable. Another trainee noted the economic benefit, saying, “We can earn money by becoming health assistants then it will bring financial prosperity to our family” (Participant 1), emphasizing how financial support can uplift their households. This statement reflects a significant opportunity for Rohingya families amid rising financial challenges stemming from recent humanitarian aid restrictions. A community leader reinforced this idea, stating, “After graduating from this program young women can apply for any job whether she is in Bangladesh or any other country” (Leader 4), demonstrating how the program can enhance mobility and livelihood opportunities.

However, despite these opportunities and expressed optimism, an expert from a community-based NGO working in the camps expressed concerns about restrictions on the employment of Rohingya within the camps. While NGOs may consider hiring skilled workers on a case-by-case basis, broader governmental employment restrictions on the Rohingya in the camps remain a significant barrier. This expert also emphasized that proficiency in English would be particularly valuable, given the lack of formal English education opportunities for Rohingya in the camps. Having skilled workers fluent in English could improve their employability, especially in roles requiring communication with international organizations. This emphasizes the importance of incorporating English language training alongside healthcare skills, ensuring that Rohingya women can fully leverage their training for better employment prospects.

### 4.3. Theme 3: Impact of the Program on the Community

The evaluation explored the potential overall impact of the HAT Program on the community, and the data revealed two impacts: (a) the direct provision of care and improved access to healthcare and (b) overall community wellbeing.

*(a)*  *Direct provision of care and improved access to healthcare*

Participants expressed that the HAT Program has the potential to improve healthcare access and overall health outcomes in the community. One major impact is addressing gender-related barriers in healthcare, as many Rohingya women feel uncomfortable discussing their medical concerns with male providers. As one trainee explained, “Women often struggle to communicate openly with male healthcare providers, but if we become health assistants, it will make it much easier for female patients to share their concerns with us comfortably and without hesitation” (Focus Group 1). By having trained female health assistants, more women may seek medical care, leading to earlier diagnoses and better treatment outcomes. Additionally, these health assistants can bridge gaps in health literacy, ensuring that patients follow medical advice. As another trainee noted, “Many people do not understand prescriptions given by the hospital, so I would like to assist them to take the correct dose” (Focus Group 2), highlighting the importance of clear and culturally aligned communication in preventing medication errors and ensuring effective treatment.

By improving individual patient care, participants noted that the program also helps address a critical shortage of healthcare personnel within the camps, particularly as NGOs face increasing budget constraints and service reductions. A community leader emphasized, “The program can reduce the scarcity of health personnel in the camp, and they will serve the people” (Leader 6), showcasing how trained health assistants can help fill essential roles. Additionally, their presence will directly benefit vulnerable groups, as another leader stated, “They will help mothers and children to get quality health services” (Leader 6). By strengthening the healthcare workforce and improving access to care, the HAT Program plays a crucial role in enhancing the overall wellbeing of the Rohingya community.

*(b)*  *Overall community wellbeing*

The participants explained that the HAT Program can significantly contribute to an overall sense of wellbeing of the community by fostering social harmony, inspiring future generations, and improving healthcare knowledge. By empowering women to serve as healthcare providers, the HAT Program can help reduce tensions within the camp, as one trainee noted, “When we become health assistants and work for the betterment of the people, it will decrease the conflict between groups” (Participant 5-Focus Group 1). Additionally, the visibility of trained women in healthcare roles may encourage others to pursue similar opportunities, gradually breaking down cultural barriers to education and employment for Rohingya girls and women. As another trainee explained, “The batch that will come after us will be inspired to come by seeing us. In this way, obstacles will be overcome gradually” (Focus group 1). Beyond individual empowerment, the HAT Program strengthens the entire community by fostering a culture of knowledge-sharing and support. A community leader emphasized this impact stating, “They will bring many benefits; they will learn themselves and educate others. Our community will benefit from this training as they will work for our community” (Leader 1). By equipping women with essential skills and creating role models within the camps, this initiative contributes to a healthier, more cohesive, and resilient Rohingya refugee community.

### 4.4. Theme 4: The Potential to Expand the HAT Program

Our evaluation also uncovered the importance of formally integrating the HAT program within existing health coordination mechanisms in the camps. An advisor from a local NGO operating clinics and schools emphasized that the HAT program should be linked to the Bangladesh Rohingya Community Health Working group (CHWG), a UNHCR-led, multi-agency coordination platform that oversees community health worker (CHW) strategies across the camps. The CHWG plays a central role in coordinating community-based surveillance, health promotion, outbreak response (e.g., COVID-19, dengue, diphtheria), and first aid training, with technical leadership from the WHO and implementation support from partners such as the Bangladesh Red Crescent Society and international NGOs [2]. Formal alignment with the CHWG would help ensure that HAT-trained health assistants are integrated into existing referral pathways, surveillance systems, and emergency response structures, while avoiding duplication of efforts and strengthening legitimacy within the humanitarian health architecture.

Establishing this connection is particularly important given ongoing challenges faced by Rohingya CHWs, including limited formal recognition, movement restrictions, and constrained pathways for skills accreditation and employment [32]. While some stakeholders viewed the HAT Program as a potential step toward more advanced health roles, such as midwifery, discussions with a midwife program coordinator indicated that formal integration into midwifery training remains highly challenging due to national policy constraints, including requirements related to Bangladeshi nationality and completion of formal secondary education, criteria that most Rohingya girls do not meet. In this context, linking the HAT Program to the CHWG offers a more realistic and system-aligned pathway for strengthening women’s participation in community health, by aligning training standards, supporting supervision and continuing education, and strengthening coordination with sexual and reproductive health services, nutrition programming, and gender-based violence referral pathways already operating in the camps. As such, formal engagement with the CHWG should be considered a key recommendation for the future sustainability, scalability, and system-level impact of the HAT Program. Such alignment could also support future training models by situating HAT graduates within recognized community health structures.

## 5. Discussion

The situation for Rohingya refugees in Bangladesh is worsening by the day. The security situation has also deteriorated sharply in recent years [33]. Increased instability along the Myanmar-Bangladesh border, rising violent crime by armed groups, and a surge in human trafficking of women and girls pose severe threats to their safety [1,34,35,36]. The recent arrival of more refugees from Myanmar has coincided with a significant decline in international humanitarian funding [2,37]. Since the COVID-19 pandemic, aid for the Rohingya has dwindled, and the 2025 closure of the United States Agency for International Development (USAID)—which previously contributed 55% of all foreign aid for Rohingya refugees in Bangladesh—has further exacerbated the crisis [34,38,39]. And their plight remains dire. More than seven years after their displacement from Myanmar, there is no viable pathway for their safe return, leaving them in a state of perpetual limbo in overcrowded refugee camps [4]. Meanwhile, the population continues to grow as families expand, placing additional strain on an already overstretched humanitarian system. This growth exacerbates the demand for essential services like healthcare, education, and protection, which are underfunded and insufficient to meet the Rohingya community’s shifting needs.

It is in this context that the HAT Program stands out as a vital initiative addressing multiple systemic gaps. It is an innovative way of utilizing the existing resources provided by the Hope Foundation, including a field maternity hospital and a midwifery school, to create a tailored training program that could benefit young Rohingya women and the community within the camps. By training young Rohingya women to provide culturally appropriate healthcare services, the HAT Program not only enhances maternal and neonatal health outcomes but also builds community trust in the healthcare system. Research consistently demonstrates that care provided by individuals from the same cultural and linguistic background as patients fosters better communication, improves patient satisfaction, and enhances the overall quality of care and health outcomes for care recipients. This has been observed in similar contexts, such as programs implemented for Syrian refugees in Turkey and Rohingya refugees in Milwaukee, USA, where culturally sensitive healthcare delivery improved health outcomes and community well-being [40,41].

The findings of this study align with global evidence supporting the role of community-based training programs in refugee settings [33]. Training local community members as healthcare providers ensures the sustainability of care, particularly in resource-constrained and politically restrictive settings. For example, programs by Médecins Sans Frontières in South Sudan and Sierra Leone successfully trained local healthcare workers to address critical skill gaps during crises [42]. Similarly, the Qatar Red Crescent Society’s initiatives for Gaza practitioners illustrate the long-term potential of training programs in conflict settings [43]. These global benchmarks underscore the relevance and scalability of the HAT Program.

However, challenges persist. Despite their enhanced skills and certifications, employment limitations on Rohingya refugees in Bangladesh may impede the economic opportunities for health assistant graduates. While some NGOs have the capacity to employ these graduates on a case-by-case basis, systemic barriers such as the lack of formal recognition for refugee-acquired qualifications hinder broader workforce integration [44]. These limitations echo findings from similar initiatives in Tanzania, where vocational training programs for refugees struggled to translate skills into economic returns [45]. Nevertheless, the social and empowerment benefits observed in these programs are significant and align with our findings.

The HAT Program’s broader societal impact is also notable. By integrating trained health assistants into the healthcare ecosystem, the initiative not only improves access to care but also serves as a model for community-driven healthcare solutions. The HAT Program fosters leadership, builds trust in the healthcare system, and promotes gender equity by empowering young women to take on active, respected roles within their communities. This aligns with evidence from community health worker programs globally, which have demonstrated positive spillover effects on community health literacy, economic development, and social cohesion [46].

While the HAT Program demonstrates promise as a community-based capacity-building initiative, its sustainability and scalability are shaped by broader structural and system-level factors beyond the scope of this evaluation. These include the need for alignment with existing health coordination mechanisms, ongoing logistical and policy constraints, and limited formal pathways for education and employment within the refugee camp context. Rather than advocating for program expansion or funding, the findings highlight the importance of situating training initiatives such as the HAT Program within established health governance structures and coordination platforms to ensure coherence, accountability, and avoid duplication of efforts. Future program decisions would benefit from independent, outcome-focused evaluations that assess longer-term impacts and explore how community-based training initiatives can complement (not substitute) formal health systems operating in humanitarian settings.

Importantly, the findings of this study should be interpreted in light of its scope. This evaluation focused on participants’ and community leaders’ perceptions of the HAT Program, its acceptability, and its anticipated benefits, rather than on direct measurement of health outcomes or training effectiveness. As a next phase, the HAT Program could be formalized and evaluated as a structured training initiative (potentially incorporating a training-of-trainers (ToT) model) focused on community-based health promotion rather than formal professional credentialing to assess effectiveness and longer-term impact. Such an evaluation could include indicators such as changes in participants’ knowledge and skills pre- and post-training, the number of graduates who become health assistants, employment or volunteer engagement, and shifts in community-level knowledge and practice related to maternal and reproductive health. Framing the present study as a formative evaluation provides an evidence-informed foundation for these future, outcome-focused assessments.

### Study Limitations

This study has several limitations that should be considered when interpreting the findings. First, the sample size was small, reflecting the pilot and exploratory nature of the HAT Program and limiting the generalizability of results. However, the primary aim of this formative program evaluation was not statistical generalization, but rather to generate in-depth, contextually grounded insights into program acceptability, feasibility, and perceived benefits within a highly constrained humanitarian setting. From this perspective, the sample was appropriate for exploring early program dynamics and informing future program refinement and evaluation.

Second, participant selection was facilitated by the non-governmental organization implementing the program, raising the possibility of selection bias. While this community-based recruitment approach was appropriate for a feasibility-focused evaluation in a humanitarian setting, it may have favored participants who were perceived as more motivated, available, or acceptable within the community. To mitigate potential coercion or undue influence, participants were repeatedly informed that their decision to participate or not participate in the evaluation would have no impact on the services, training, or support they received through the Hope Foundation. Participation was voluntary, and all semi-structured interviews and focus group discussions were conducted with this assurance clearly communicated.

Third, although Chittagonian is closely related to the Rohingya language and widely used as a lingua franca in the camps, data collection may have benefited from the inclusion of an interviewer who was fully fluent in Rohingya and from the Rohingya community. While steps were taken to address potential interpretation challenges through bilingual transcription, contextual translation, and internal review, this linguistic and cultural distance remains a limitation. At the same time, the involvement of locally based team members with extensive experience working in Rohingya-speaking contexts supported rapport-building and culturally appropriate engagement.

Fourth, the study did not include formal pre- and post-tests to assess changes in knowledge or skills over time. Given the low literacy levels among participants and the design of the program, no standardized assessments were administered. Instead, the initial focus group discussions included questions designed to gauge trainees’ baseline understanding of the healthcare system. Additional exit focus group discussions were conducted after program completion (which was outside the scope of this manuscript), exploring trainees’ learning experiences and perceived personal and professional growth. While these qualitative data provide valuable insight into perceived learning and empowerment, they do not substitute for objective outcome measures.

Finally, the data are cross-sectional and capture perceptions at a single point in time. Longitudinal research is needed to assess the sustained impact of the HAT Program on trainees, their families, and community health outcomes. Notably, a separate evaluation of the HAT Program is currently underway, led by an independent research team and employing Rohingya-speaking interviewers. Together, these forthcoming findings will complement the present formative evaluation and contribute to a more comprehensive understanding of the program’s longer-term effectiveness and impact.

## 6. Recommendations and Conclusions

This formative evaluation demonstrates that the Health Assistant Training (HAT) Program is a culturally acceptable, community-supported, and promising capacity-building initiative within a highly constrained humanitarian setting. By equipping young Rohingya women with essential maternal and child health knowledge and skills, the program addresses critical gaps in healthcare access while simultaneously fostering women’s empowerment, trust in health services, and community resilience. While this study did not assess effectiveness or health outcomes directly, the findings offer important guidance for strengthening, sustaining, and responsibly scaling the HAT Program. Based on the study findings, the following recommendations are proposed.

### 6.1. Recommendation #1

Formal integration of the HAT Program within existing health coordination mechanisms is essential. In particular, alignment with the Bangladesh Rohingya Community Health Working Group (CHWG)—a UNHCR-led, multi-agency coordination platform—should be prioritized. Formal engagement with the CHWG would help situate HAT graduates within recognized community health governance structures, strengthen coordination with existing referral pathways and surveillance systems, and avoid duplication of efforts. Such integration would also enhance the legitimacy, sustainability, and system-level impact of the program within the broader humanitarian health architecture. Consistent with Agarwal et al.’s [3] emphasis on community-owned, system-embedded maternal health strategies, formal alignment with existing coordination mechanisms is essential to ensure sustainability and avoid parallel service structures. Similarly, evidence from Nguyen et al.’s [7] research on integrated nutrition and psychosocial programs for Rohingya refugees suggests that formal coordination and supervision structures are critical to sustaining program quality and impact over time, particularly in resource-constrained settings.

### 6.2. Recommendation #2

The HAT Program should be strengthened as a structured community-based capacity-building model rather than positioned as a pathway to formal professional credentialing. Given current national policy constraints related to citizenship, education, and employment [47], expectations regarding progression into formal midwifery or professional health roles should be managed realistically. Instead, the program’s value lies in its ability to develop trusted community-based health actors who can support health promotion, patient navigation, and maternal and child health outreach. Framing the program within this role aligns with community health worker and workforce-development models widely used in humanitarian settings. This recommendation aligns closely with broader humanitarian priorities articulated in the *Joint Response Plan* [2] that emphasizes localization through the expansion of Rohingya-led roles across sectors, including health, education, protection, and community outreach. The *Joint Response Plan* highlights both the scale of Rohingya participation already underway through training of teachers, facilitators, and volunteers. It also identified the persistent gap regarding the need for an opportunity due to funding and structural constraints. Within this context, the HAT Program represents a complementary, gender-responsive approach to Rohingya capacity building that strengthens frontline health services while creating meaningful skill-development pathways for young women.

### 6.3. Recommendation #3

Future iterations of the HAT Program could explicitly incorporate training-of-trainers (ToT) principles to enhance reach and sustainability. While not formally designed as a ToT initiative, the program already demonstrates strong potential for knowledge diffusion through households and community networks. A structured ToT approach could support selected graduates to mentor subsequent cohorts, contribute to peer education, and extend health knowledge more broadly within the camps, particularly in contexts of shrinking humanitarian resources. This kind of structured approach also fits with sector-wide efforts to reduce duplication through cross-sector collaboration and to mitigate the severe impacts of future funding decreases [10].

### 6.4. Recommendation #4

Future evaluations should move beyond feasibility and acceptability toward outcome-focused and longitudinal assessment. Subsequent research should examine changes in trainees’ knowledge and skills over time, community-level health literacy, and potential shifts in care-seeking behaviors related to maternal and reproductive health. Where feasible, mixed-methods approaches incorporating adapted, low-literacy assessment tools and longer-term follow-up would strengthen the evidence base while remaining contextually appropriate. Similar to other pragmatic evaluations in Rohingya camp settings, such approaches would strengthen causal inference while remaining feasible in humanitarian contexts [7,8].

## 7. Conclusions

In conclusion, the HAT Program represents a timely and contextually grounded response to intersecting gaps in healthcare access, education, and women’s opportunities in the Rohingya refugee camps. While structural and policy constraints continue to limit formal employment pathways, this evaluation highlights the program’s potential to strengthen community health capacity, empower young women, and contribute to more resilient and trusted healthcare systems. With strategic integration, realistic framing, and continued evaluation, the HAT Program offers a promising model for other protracted refugee and humanitarian settings facing similar constraints.

## Figures and Tables

**Table 1:** Participant Categories and Data Collection Characteristics.

Participant Category	Number of Participants	Method of Data Collection	Data Capture Technique	Language Used
HAT Trainees (female Rohingya, 14–19 years old)	9	focus group discussions	audio-recorded and transcribed	Rohingya/ Chittagonian
Community Leaders (male *majis*)	8	semi-structured interviews	audio-recorded and transcribed	Rohingya/ Chittagonian
NGO Stakeholders /Representatives	5	semi-structured interviews	detailed notes	English

## Data Availability

The datasets presented in this article are not readily available because of the sensitive nature of the topic and the precarious status of the evaluation participants. Requests to access datasets should be directed to alnaji.nada@gmail.com.
